# WFUMB-Recommended Versus Manufacturer-Optimized Protocols for Ultrasound-Guided Attenuation Parameter in Hepatic Steatosis Assessment: A Retrospective Single-Vendor Multicenter Study

**DOI:** 10.3390/jcm15218373

**Published:** 2026-10-27

**Authors:** Hidekatsu Kuroda, Takuma Oguri, Naohisa Kamiyama, Hidenori Toyoda, Satoshi Yasuda, Kento Imajo, Yasuaki Suzuki, Katsutoshi Sugimoto, Tomoyuki Akita, Junko Tanaka, Yutaka Yasui, Masayuki Kurosaki, Namiki Izumi, Atsushi Nakajima, Yudai Fujiwara, Tamami Abe, Keisuke Kakisaka, Takayuki Matsumoto, Takashi Kumada

**Affiliations:** 1 Division of Gastroenterology and Hepatology, Department of Internal Medicine, School of Medicine, Iwate Medical University, 2-1-1 Idaidori, Yahaba, Iwate 028-3695, Japankeikaki@iwate-med.ac.jp (K.K.);; 2 Ultrasound General Imaging, GE HealthCare, Hino 191-8503, Japan; 3 Department of Gastroenterology and Hepatology, Ogaki Municipal Hospital, Ogaki 503-8502, Japan; 4 Department of Gastroenterology, Shin-Yurigaoka General Hospital, Kawasaki 215-0026, Japan; 5 Department of Gastroenterology, Nayoro City General Hospital, Nayoro 096-8511, Japan; 6 Department of Gastroenterology and Hepatology, Tokyo Medical University, Tokyo 160-8402, Japan; 7 Department of Epidemiology, Infectious Disease Control, and Prevention, Hiroshima University Institute of Biomedical and Health Sciences, Hiroshima 734-8551, Japan; 8 Department of Gastroenterology and Hepatology, Musashino Red Cross Hospital, Musashino 180-8610, Japan; 9 Department of Gastroenterology and Hepatology, Yokohama City University Graduate School of Medicine, Yokohama 236-0004, Japan; 10 Department of Nursing, Gifu Kyoritsu University, Ogaki 503-8550, Japan

**Keywords:** hepatic steatosis, ultrasound-guided attenuation parameter, WFUMB, MRI-PDFF, MASLD, attenuation coefficient, measurement protocol, single-vendor, multicenter study

## Abstract

**Background/Objectives**: The ultrasound-guided attenuation parameter (UGAP) is conventionally measured at a 4 cm start depth from the skin with a 4 cm region of interest (ROI), whereas 2024 World Federation for Ultrasound in Medicine and Biology (WFUMB) guidance recommends 2 cm from the liver capsule with a 3 cm ROI. We compared these two ROI configurations, applied to the same archived acquisitions, against MRI-derived proton density fat fraction (MRI-PDFF). **Methods**: This retrospective study included 565 patients with chronic liver disease from four of six participating Japanese centers at which raw data had been stored. Conventional UGAP was measured in real time; WFUMB-based values were calculated retrospectively from the same acquisitions, without real-time operator optimization. Performance was assessed by receiver operating characteristic analysis and the Obuchowski index, with integrated discrimination improvement (IDI) and net reclassification improvement (NRI) as secondary analyses. **Results**: Areas under the curve did not differ significantly at any threshold (≥S1: 0.92 [95% CI: 0.90–0.94] vs. 0.92 [0.89–0.94]; *p* = 0.07–0.23). Conventional UGAP correlated slightly more strongly with MRI-PDFF (ρ = 0.83 vs. 0.81; Δρ = 0.02; *p* < 0.01) and had a higher Obuchowski index (0.92 vs. 0.91; *p* = 0.04); both differences were statistically significant but of doubtful clinical relevance, and secondary IDI/NRI analyses were directionally consistent but imprecise. Body mass index was the strongest independent predictor of the inter-protocol difference. **Conclusions**: Neither ROI configuration showed a clinically meaningful advantage on this single-vendor platform. Small differences favoring the manufacturer-optimized configuration were not uniform across centers and may largely reflect acquisition asymmetry. No equivalence margin was prespecified, so these data show no demonstrable advantage for either configuration rather than establishing equivalence and require confirmation in a prospective paired study.

## 1. Introduction

Metabolic dysfunction-associated steatotic liver disease (MASLD) affects approximately 30% of the general population and is closely associated with metabolic and cardiovascular diseases through mechanisms such as insulin resistance, metabolic dysfunction, and chronic inflammation [1,2,3,4]. Therefore, accurate and widely accessible methods for quantifying hepatic steatosis are of substantial clinical importance.

The ultrasound attenuation coefficient shows a strong correlation with MRI-derived proton density fat fraction (MRI-PDFF; ρ = 0.74–0.83) [5,6]. Accordingly, several ultrasound-based technologies have been developed for clinical use, including the ultrasound-guided attenuation parameter (UGAP; GE HealthCare, Chicago, IL, USA). Previous studies have reported area under the receiver operating characteristic curve (AUC) values exceeding 0.90 for detecting steatosis [7,8], with initial validation demonstrating excellent diagnostic performance in chronic liver disease [9,10,11]. Attenuation-based techniques from other manufacturers have likewise shown high diagnostic accuracy against MRI-PDFF and histology [12], indicating that the principle is robust across platforms.

However, no universally harmonized measurement protocol exists. The UGAP employs fixed settings (4 cm start depth from the skin surface, 4 cm region of interest [ROI]) based on manufacturer optimization [8], whereas the 2024 WFUMB guidance recommends a start depth of 2 cm from the liver capsule with a 3 cm ROI [13]. Previous studies have reported stronger correlations with MRI-PDFF at deeper measurement settings [14], suggesting that acquisition parameters may affect diagnostic accuracy. The WFUMB recommendations are based on general physical principles, and the guidance acknowledges that device-specific optimization may differ [13]. This lack of standardization may lead to inconsistent steatosis grading across institutions. How the WFUMB ROI configuration performs when applied to UGAP has not been examined.

In this retrospective analysis of a prospective multicenter cohort, we compared the conventional (4 cm/4 cm) and WFUMB-based (2 cm/3 cm) ROI configurations applied to the same archived UGAP acquisitions in patients with chronic liver disease, using MRI-PDFF as the reference standard, and explored the factors associated with the difference between them. Because the WFUMB-based values were reconstructed retrospectively, the study was designed as a comparison of ROI configurations within archived acquisitions rather than as a head-to-head trial of two prospectively acquired protocols.

## 2. Materials and Methods

### 2.1. Study Design and Population

The study protocol was approved by the institutional review board of each participating center, and written informed consent was obtained from all participants. The study was conducted in accordance with the Declaration of Helsinki 2013, registered in the UMIN Clinical Trials Registry (UMIN000041196), and reported according to the Strengthening the Reporting of Observational Studies in Epidemiology (STROBE) statement.

This study was a secondary analysis of a prospective multicenter study conducted at six Japanese liver centers to evaluate the diagnostic performance of UGAP for hepatic steatosis [8].

Patients were eligible if they were aged ≥18 years, had known or suspected chronic liver disease, and had both conventional and WFUMB-based UGAP measurements available during the same examination session. The exclusion criteria were contraindications for MRI, incomplete imaging data, and technical failure of the ultrasound examination. Of the 1010 patients in the original cohort, 445 were excluded because their raw data were not stored during the original examination (*n* = 430), were of insufficient quality for retrospective reanalysis (*n* = 13), or were inadvertently lost (*n* = 2). Storage of raw data was not a prespecified element of the original study protocol, which was designed to evaluate real-time UGAP. Because each raw data set occupies substantial storage space and prolongs the examination, raw data were saved at the discretion of the sonographer when storage capacity and examination time permitted, and at two of the six centers, storage was not incorporated into the routine workflow at all. Availability was therefore governed by the workflow of each center rather than by any assessment of individual patients. To assess the potential for selection bias, we compared included and excluded patients overall and, separately, within the centers that contributed analyzable data (Supplementary Table S2) and examined inclusion numbers, recruitment period and diagnostic performance by center (Supplementary Table S5).

### 2.2. UGAP Measurements

All examinations were performed using a LOGIQ E10 system (GE HealthCare, Chicago, IL, USA) equipped with a C1-6 convex transducer (1–6 MHz). The patients fasted for at least 4 h prior to examination. Attenuation coefficient measurements (dB/cm/MHz) were obtained in the supine position using an intercostal approach targeting the right hepatic lobe, with care taken to avoid the major vessels and biliary structures.

One or two dedicated sonographers at each center performed all the UGAP examinations. Each sonographer completed UGAP-specific training and had experience with more than 1000 conventional ultrasound examinations in accordance with the European Federation of Societies for Ultrasound in Medicine and Biology guidelines and recommendations [15], along with more than 50 supervised two-dimensional shear wave elastography examinations.

**Figure 1:**
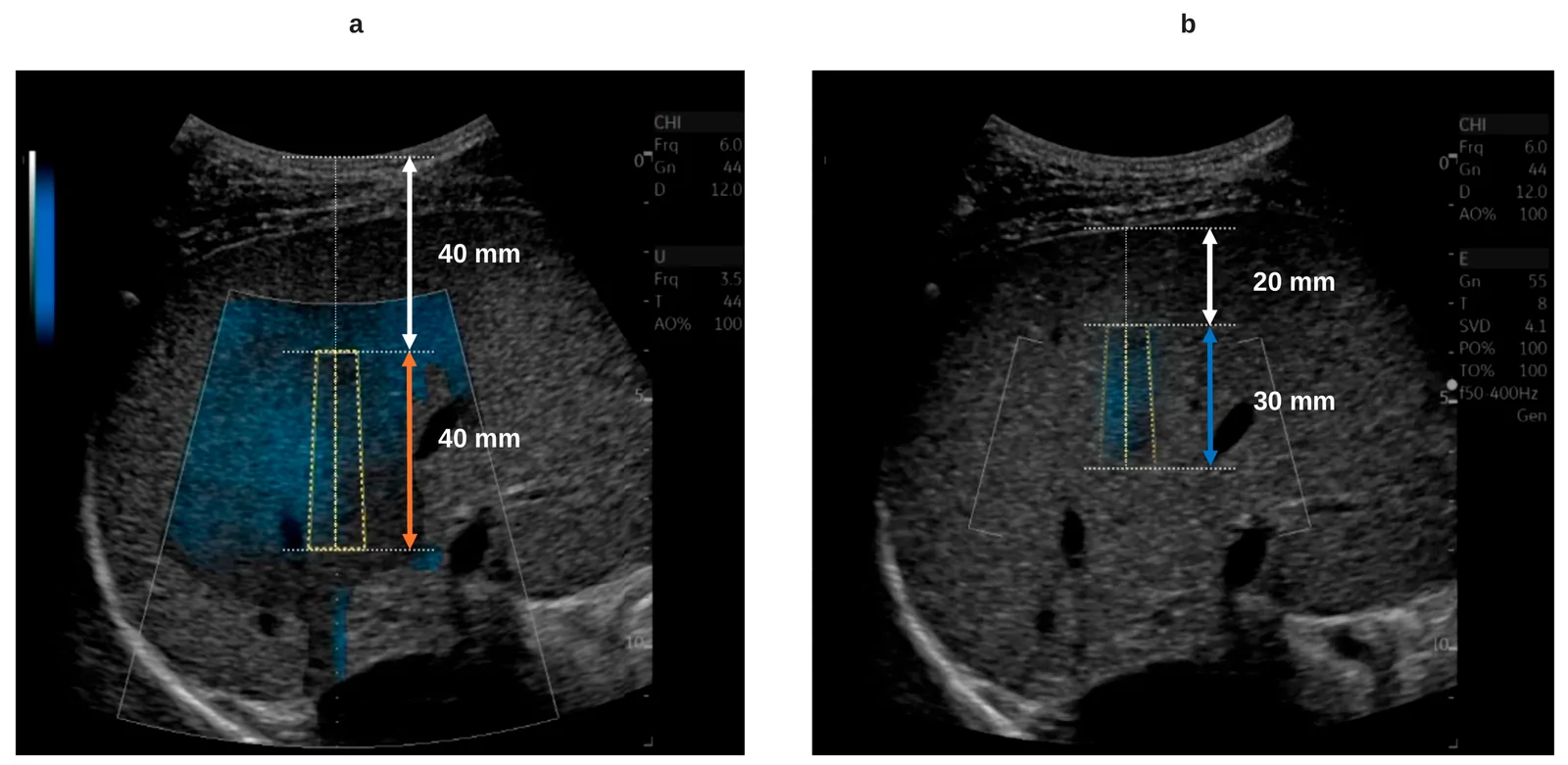
Measurement screens of (**a**) conventional UGAP, acquired with the manufacturer-optimized settings (start depth 4 cm from the skin surface; 4 cm ROI), and (**b**) WFUMB-based UGAP, reconstructed with the WFUMB-recommended settings (start depth 2 cm from the liver capsule; 3 cm ROI). In both panels the white arrow indicates the start depth, and the coloured arrow (orange in (**a**), blue in (**b**)) indicates the ROI length.

Conventional UGAP (4 cm/4 cm) was measured in real time using manufacturer-optimized settings (start depth, 4 cm; ROI length, 4 cm) (Figure 1a). A minimum of five valid measurements were obtained and the median value recorded. In the conventional UGAP protocol, the nominal measurement region spans 4 cm in depth starting from 4 cm below the skin surface (i.e., 4–8 cm depth). However, this function incorporates an automated fine-adjustment algorithm for ROI position and size. When interfering structures such as the diaphragm are detected within the measurement region, or when the signal-to-noise ratio is insufficient due to inadequate signal components, the system automatically performs minor adjustments to the ROI position and length along the depth axis to avoid these regions and ensure reliable attenuation estimation. Raw data from each acquisition were stored for subsequent analyses.

The WFUMB-based UGAP (2 cm/3 cm) was calculated retrospectively from stored raw data by repositioning the ROI according to WFUMB recommendations (start depth 2 cm from the liver capsule, ROI length 3 cm) (Figure 1b) [13]. The offline software used for ROI repositioning was implemented by the manufacturer’s engineers (T.O. and N.K.), who had no access to clinical or MRI data. This analysis was performed centrally by one of two hepatologists (H.K. or T.A.) blinded to all clinical and imaging data. The number of stored raw data frames ranged from 5 to 10 per patient, and a minimum of five valid WFUMB-based measurements were successfully obtained in all cases. This approach enabled direct comparison of both ROI configurations using identical acquisitions, thereby eliminating variability in probe positioning or patient respiration; however, it also meant that the WFUMB-based values were obtained without the real-time operator optimization (probe adjustment, selection of respiratory phase, rejection of poor frames) and automated fine adjustment that were available for the conventional measurements. This asymmetry is addressed in the Discussion Section.

### 2.3. MRI-PDFF

MRI was performed using a 3.0-T system (Discovery MR750w 3.0T; GE HealthCare, Waukesha, WI, USA). Proton density fat fraction (PDFF) was measured using a multi-echo Dixon method (IDEAL-IQ sequence) with the following parameters: repetition time, 6.6 ms; echo times, 6 echoes ranging from 0.9 to 4.8 ms; field of view, 44 cm; matrix, 160 × 160; slice thickness, 8.0 mm; flip angle, 1°; bandwidth, 111.11 kHz; acquisition time, 19 s breath-hold. Consistent with prior UGAP validation studies [8,9,10,11], a single cubic ROI (20 × 20 × 20 mm^3^) was placed in hepatic segment VII or VIII to avoid vessels and artifacts; this corresponded to the ultrasound measurement site in the right hepatic lobe.

The PDFF analysis was performed by hepatologists at each institution (each with >3 years of MRI interpretation experience) who were blinded to the UGAP results. Steatosis grades were defined according to previously validated thresholds [16]: S0 (PDFF < 5.2%), S1 (5.2% ≤ PDFF < 11.3%), S2 (11.3% ≤ PDFF < 17.1%), and S3 (PDFF ≥ 17.1%).

### 2.4. Assessment of Hepatic Fibrosis

Magnetic resonance elastography (MRE) was performed in the same MRI session on the same 3.0-T system. Continuous 60 Hz mechanical waves were generated by a passive driver on the anterior chest wall, and a two-dimensional spin-echo echo-planar sequence was used, as described previously [16]. Regions of interest included liver parenchyma only, avoiding hepatic edges and large vessels, and the mean of three regions was recorded as the shear modulus in kilopascals. Fibrosis was staged using the MRE cutoffs validated against histology in the same study as our steatosis thresholds [16]: F0 (<2.5 kPa), F1 (2.5–3.3 kPa), F2 (3.4–4.7 kPa), F3 (4.8–6.6 kPa) and F4 (≥6.7 kPa). MRE was available in 561 of the 565 patients.

Two-dimensional shear wave elastography (2D-SWE) was performed on the same LOGIQ E10 system in the same session as UGAP, using an intercostal approach to the right hepatic lobe with the patient supine and in breath-hold. Five valid measurements were obtained per patient, and the median was recorded; acquisitions were reliable when the interquartile range/median ratio was <30%. 2D-SWE was available in 559 patients. The Fibrosis-4 (FIB-4) index was calculated as (age × AST)/(platelet count [10^9^/L] × √ALT).

### 2.5. Clinical and Laboratory Variables

Clinical and laboratory data were collected on the day of ultrasound examination. Body mass index (BMI) was calculated as weight divided by height squared. Etiology was classified based on clinical history, serological markers, and histological findings where available: hepatitis B virus (HBsAg positive), hepatitis C virus (anti-HCV and HCV-RNA positive), MASLD (hepatic steatosis with metabolic risk factors in the absence of secondary causes), alcoholic liver disease (alcohol intake > 30 g/day for men or >20 g/day for women), and others. The albumin–bilirubin (ALBI) score was calculated using the formula: (log10 bilirubin [μmol/L] × 0.66) + (albumin [g/L] × −0.085), with laboratory values obtained on the day of examination.

### 2.6. Statistical Analysis

Normality was assessed using the Shapiro–Wilk test. Data were presented as means ± SDs for normally distributed variables or as medians [interquartile ranges] for non-normally distributed variables. PDFF values were log-transformed before correlation analysis. Agreement between protocols was evaluated using Bland–Altman analysis [17] and the intraclass correlation coefficient (ICC; two-way mixed model, single measures, absolute agreement). Proportional bias was assessed by regressing the difference on the mean. Correlation with MRI-PDFF was assessed using Spearman’s rank correlation coefficient, with between-protocol differences compared using Steiger’s Z-test.

The primary comparison of diagnostic performance was based on receiver operating characteristic analysis with DeLong’s test [18] at each steatosis threshold, and on the Obuchowski index for overall discrimination across ordinal steatosis grades [19], computed as the weighted average of the pairwise areas under the curve for all pairs of grades, weighting each pair by the absolute difference in grade and by the number of discordant pairs. Integrated discrimination improvement (IDI) and category-free net reclassification improvement (NRI), with WFUMB-based UGAP as the reference [20], were prespecified as secondary supportive analyses because category-free NRI is known to yield inflated values and high false-positive rates [21]; both are reported with asymptotic 95% confidence intervals and *p*-values. Because the asymptotic variance of the category-free NRI is known to be anti-conservative, we additionally computed percentile bootstrap intervals in which patients were resampled with replacement 2000 times, and both logistic models were re-estimated within every resample, so the uncertainty of model estimation is also propagated; these intervals are reported alongside the asymptotic intervals in the Results. Bootstrap internal validation (2000 resamples) was performed using Harrell’s optimism-correction method to obtain optimism-corrected concordance indices (C-indices) and optimism-corrected R^2^ for the multiple regression model.

Factors affecting inter-protocol differences were assessed using the Mann–Whitney U test or Kruskal–Wallis test for categorical variables and Spearman correlation for continuous variables. Independent predictors were identified using multiple linear regression with standardized coefficients. Collinearity was assessed using the variance inflation factor (VIF), and models were considered acceptable when all VIF values were below 5. Because BMI and skin-to-capsule distance (SCD) were strongly correlated (ρ = 0.81), the primary model included BMI; as sensitivity analyses, the model was refitted with SCD in place of BMI and with both variables entered simultaneously. To keep the models comparable, all three were fitted on the same complete-case set.

Three additional analyses assessed whether hepatic fibrosis confounded the comparison. First, correlation with log MRI-PDFF and diagnostic performance were compared within MRE-based fibrosis strata (F0–1, F2–3 and F4) using Steiger’s Z-test and DeLong’s test. Second, multivariable linear regression with the attenuation coefficient as the dependent variable tested whether MRE was independently associated with attenuation after adjustment for log MRI-PDFF, BMI and SCD; all variables were standardized. Third, the difference between protocols was compared across strata by the Kruskal–Wallis test and correlated with MRE, 2D-SWE and FIB-4. Diagnostic performance was additionally compared within subgroups defined by skin-to-capsule distance (<20 vs. ≥20 mm and <25 vs. ≥25 mm) using the same tests. To assess center effects, Spearman ρ with log MRI-PDFF and the AUC for ≥S1 steatosis were calculated and compared between protocols within each center; the inter-protocol difference was compared across centers by the Kruskal–Wallis test; and center was added as a fixed effect to the multivariable model of the inter-protocol difference (F-test for the joint contribution of center).

Sample size estimation based on the original prospective multicenter study (steatosis prevalence: 65%, AUC: 0.89–0.91) indicated that approximately 500 patients were required for 95% confidence intervals within ±0.02. Statistical significance was set at *p* < 0.05. Statistical analyses were performed using EZR version 1.53 with the pROC and Hmisc packages.

### 2.7. Use of Generative Artificial Intelligence

Generative artificial intelligence (Claude, Opus 5; Anthropic, San Francisco, CA, USA) was used solely to assist with language editing of the manuscript text, namely to improve the grammar, clarity and readability of passages drafted by the authors. It was not used to design the study, to generate, analyze or interpret the data, to produce the figures or tables, or to draft scientific content or conclusions. All authors reviewed the edited text in full.

## 3. Results

### 3.1. Patient Characteristics

**Figure 2:**
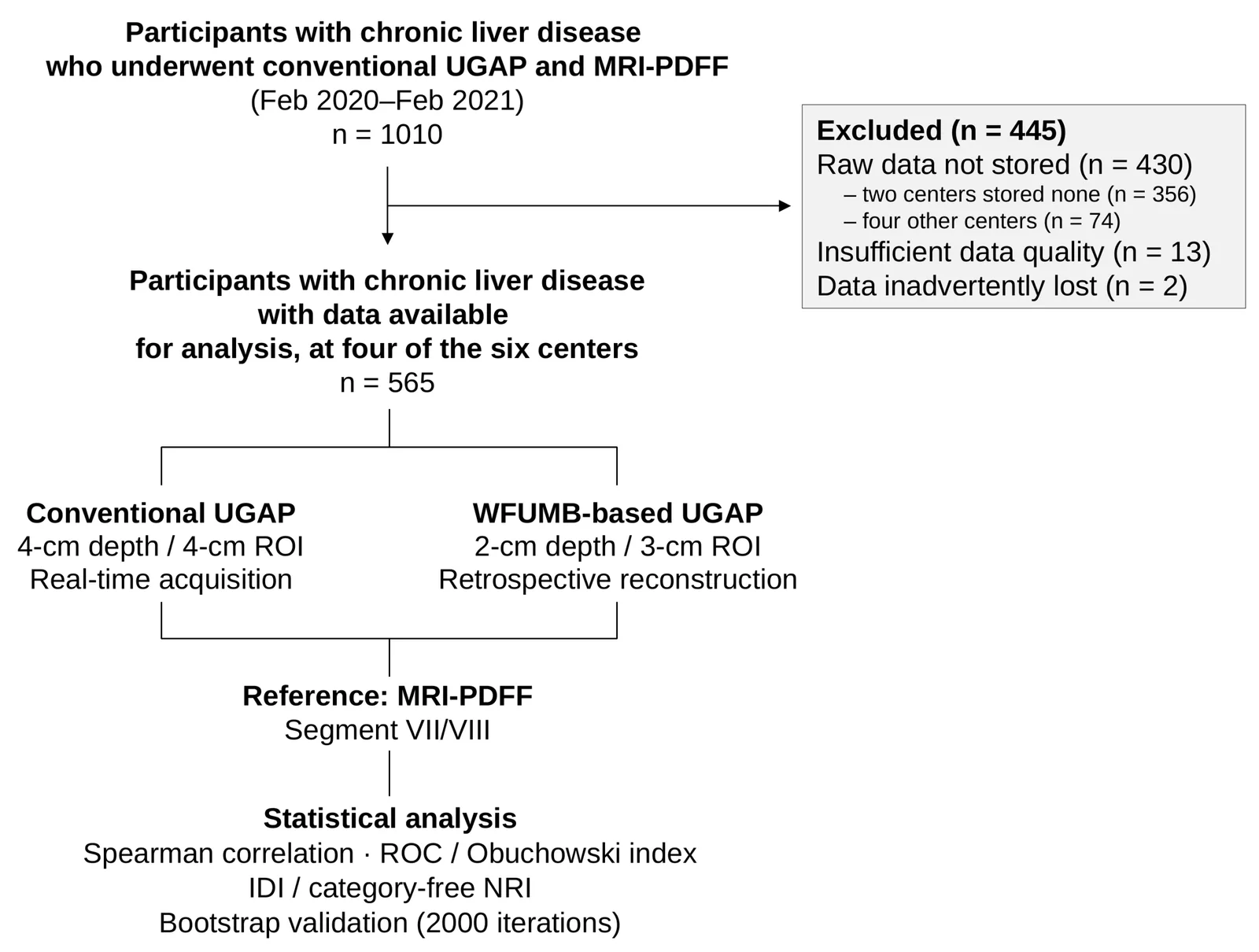
Flow chart of eligible patients with chronic liver disease.

A total of 565 patients with analyzable stored raw data were included in the final analysis (Figure 2). The median age was 64 years, and 302 patients (53.5%) were male. The predominant etiology was MASLD (*n* = 293, 51.9%), followed by hepatitis B (*n* = 89, 15.8%) and hepatitis C (*n* = 67, 11.9%). Median BMI was 25.4 [22.9–28.2] kg/m^2^. The distribution of steatosis grades was 38.6% S0 (*n* = 218), 25.8% S1 (*n* = 146), 15.6% S2 (*n* = 88), and 20.0% S3 (*n* = 113). Within this analyzable cohort, at least one valid attenuation coefficient value per protocol was obtained in all 565 patients; this figure refers to the selected cohort with stored raw data and should not be interpreted as an estimate of overall technical feasibility. The number of valid WFUMB-based measurements ranged from 5 to 10 per patient, meeting the WFUMB-recommended minimum in all cases. Patient characteristics are summarized in Table 1. The distribution of MRE-based fibrosis stages was F0 in 205 patients (36.5%), F1 in 158 (28.2%), F2 in 110 (19.6%), F3 in 58 (10.3%) and F4 in 30 (5.3%); MRE was unavailable in four patients.

Compared with the 445 excluded patients, the analysis cohort was similar in age, sex and BMI but had a lower median MRI-PDFF (7.4% vs. 9.3%; *p* < 0.001) and a greater subcutaneous fat thickness (19.8 vs. 17.6 mm; *p* < 0.001), although the distribution of steatosis grades did not differ (*p* = 0.079) (Supplementary Table S2, Panel A). Body habitus in this comparison is the measurement recorded in the parent study, the only one available for the excluded patients; the skin-to-capsule distance used in the present analyses was measured on the stored images and exists only for the analysis cohort. Center-specific numbers are given in Supplementary Table S5. Analyzable raw data were available at four of the six participating centers, where 78–92% of registered patients could be reanalyzed (286/310, 153/196, 91/106 and 35/42); at the remaining two centers, raw data had not been stored during the original examinations, so none of their 356 registered patients could be included. These two centers therefore accounted for 356 of the 445 exclusions (80%). Within the four contributing centers, included and excluded patients did not differ significantly in age, sex, BMI, subcutaneous fat thickness, MRI-PDFF or steatosis grade (all *p* ≥ 0.07; Supplementary Table S2, Panel B), so the differences in the overall comparison are attributable to the case mix of the two non-contributing centers; no evidence of selection at the patient level was found, although selection on unmeasured characteristics cannot be excluded. Within each center, AUCs for ≥S1 steatosis did not differ significantly between protocols except at the largest center (0.92 vs. 0.90; *p* = 0.01), and the direction of the difference in correlation with MRI-PDFF was not uniform: it favored the conventional protocol at the largest center (ρ = 0.820 vs. 0.781; *p* < 0.001) and the WFUMB-based protocol at the smallest (ρ = 0.781 vs. 0.870; *p* = 0.03), with no significant difference at the other two. The median inter-protocol difference ranged from 0.010 to 0.040 dB/cm/MHz and differed across centers (Kruskal–Wallis *p* < 0.001), being largest at the two centers with the highest median BMI (27.5 and 28.3 kg/m^2^).

### 3.2. Comparison of Attenuation Coefficient Values

**Figure 3:**
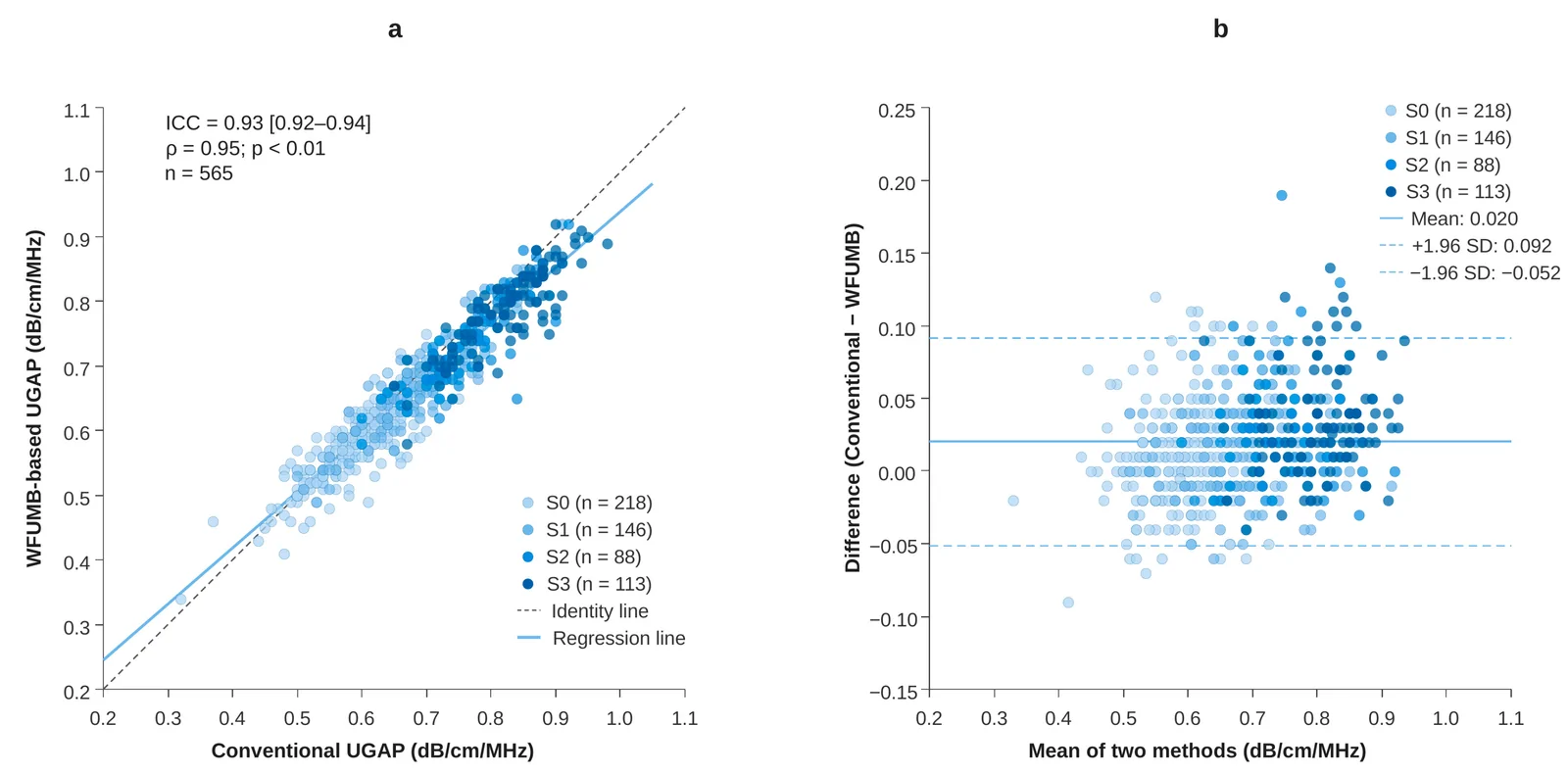
Relationship between conventional UGAP and WFUMB-based UGAP (**a**). Bland–Altman agreement between conventional UGAP and WFUMB-based UGAP (**b**).

Conventional UGAP yielded significantly higher values than WFUMB-based UGAP (median, 0.69 [IQR, 0.60–0.78] vs. 0.67 [0.59–0.75] dB/cm/MHz; *p* < 0.01). The two protocols showed a strong positive correlation (ρ = 0.95; *p* < 0.01; Figure 3a). Bland–Altman analysis revealed a median difference of 0.02 dB/cm/MHz with 95% limits of agreement ranging from −0.05 to 0.09 dB/cm/MHz (Figure 3b). A proportional bias was present (r = 0.28; *p* < 0.01), indicating greater differences at higher attenuation values. Despite this systematic bias, overall agreement was excellent (ICC = 0.93 [95% CI: 0.92–0.94]).

### 3.3. Correlation with MRI-PDFF

**Figure 4:**
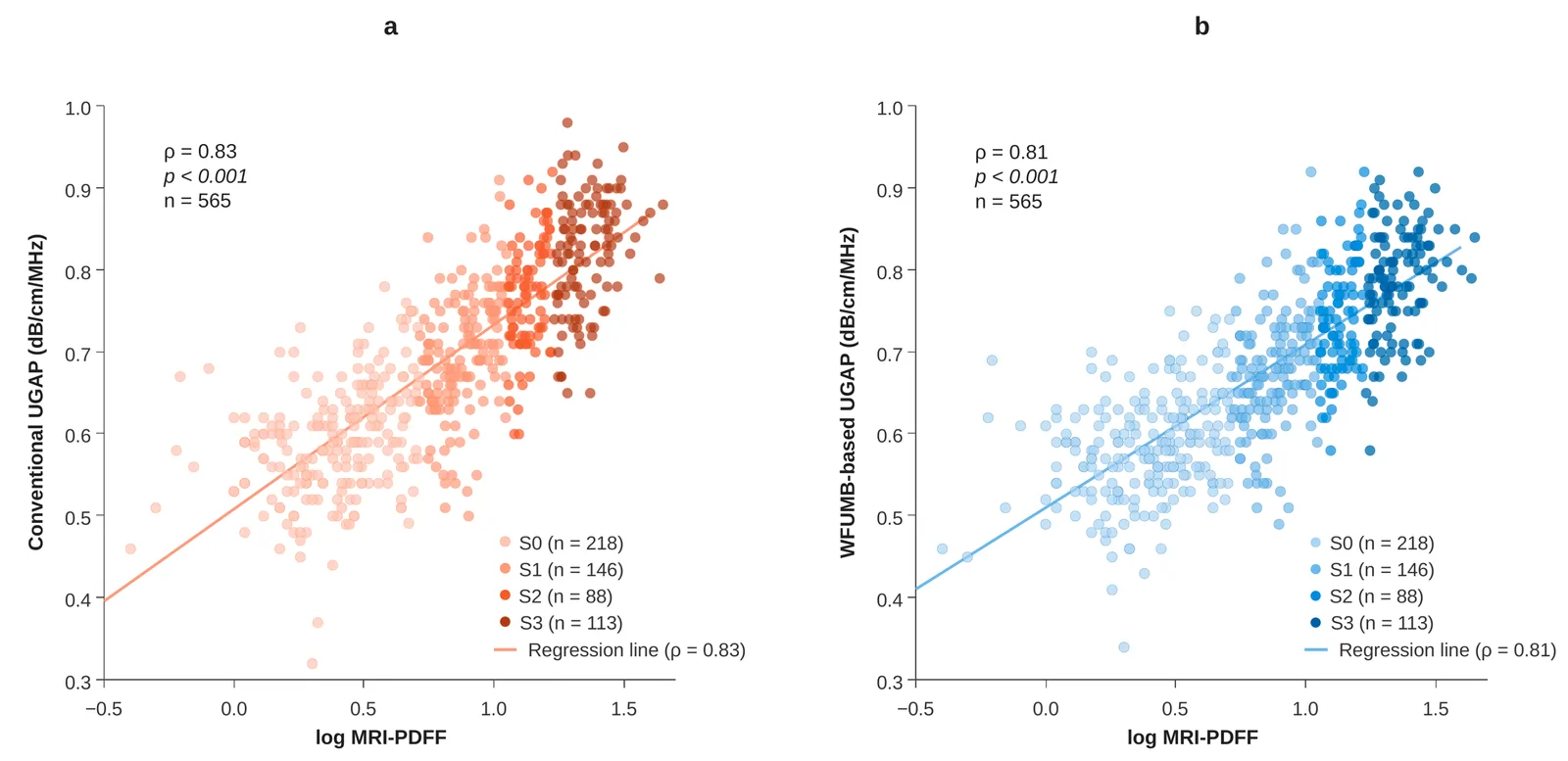
Correlation with MRI-PDFF: (**a**) conventional UGAP; (**b**) WFUMB-based UGAP.

Conventional UGAP demonstrated a slightly stronger correlation with log MRI-PDFF than WFUMB-based UGAP (ρ = 0.83 vs. 0.81; Figure 4). The difference between correlation coefficients was statistically significant (Steiger’s Z = 3.27; *p* < 0.01), although its absolute magnitude was small (Δρ = 0.02).

### 3.4. Diagnostic Performance

**Figure 5:**
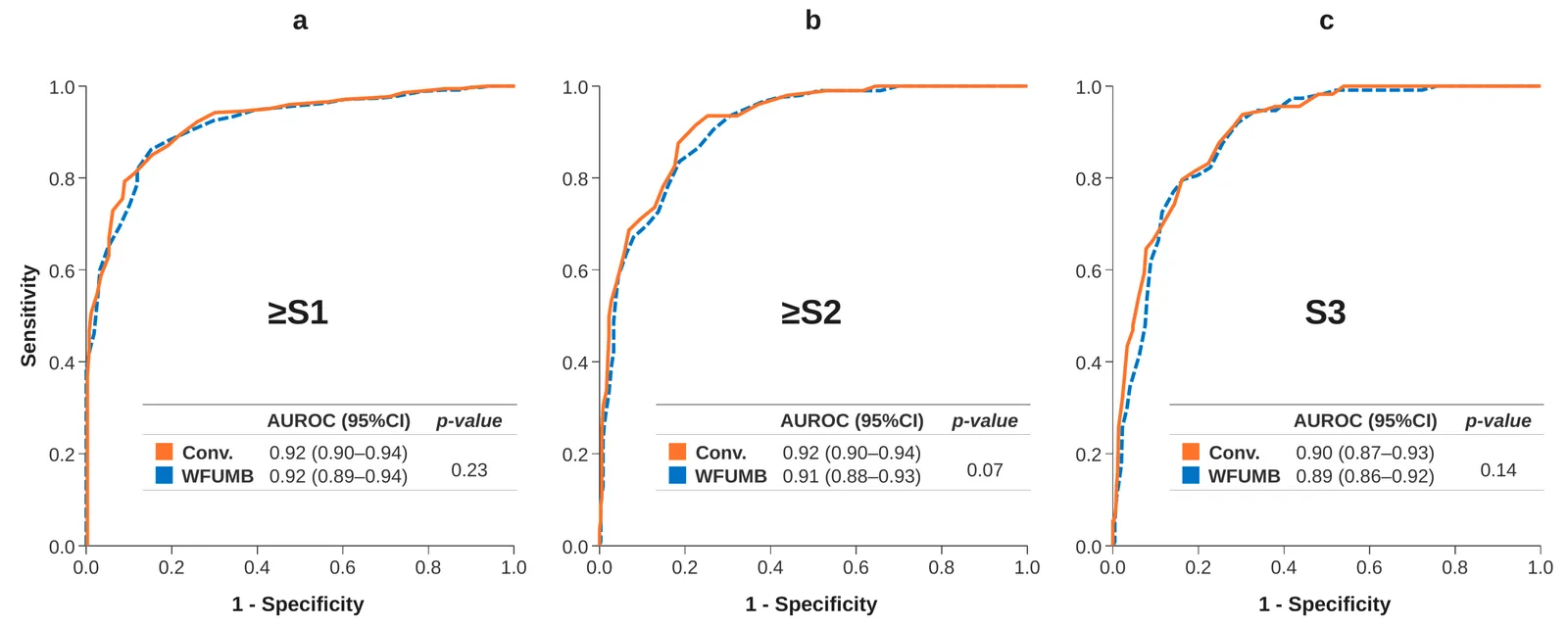
Comparison of the diagnostic performance of conventional UGAP and WFUMB-based UGAP for steatosis. ≥S1 (**a**), ≥S2 (**b**), and S3 (**c**).

ROC analysis showed closely similar performance for the two protocols across all steatosis thresholds (Table 2; Figure 5). For detection of any steatosis (≥S1), conventional UGAP achieved an AUC of 0.92 (95% CI: 0.90–0.94) compared with 0.92 (95% CI: 0.89–0.94) for WFUMB-based UGAP (*p* = 0.23). AUCs were likewise similar at ≥S2 (0.92 vs. 0.91; *p* = 0.07) and S3 (0.90 vs. 0.89; *p* = 0.14); values were numerically higher for conventional UGAP at every threshold, but none of these differences reached statistical significance.

To assess discrimination ability across steatosis grades, we calculated the Obuchowski index (Table 3). The Obuchowski index was statistically significantly higher for conventional UGAP (0.92 vs. 0.91; *p* = 0.04), an absolute difference of 0.01. In the secondary supportive analyses, IDI and category-free NRI were positive at all thresholds with WFUMB-based UGAP as the reference (IDI 0.021–0.042; NRI 0.24–0.53), showing consistent directionality favoring conventional UGAP (Table 3). Their precision was limited: the IDI did not reach significance at the ≥S1 threshold (*p* = 0.07), the threshold at which the two AUCs were closest, and bootstrap intervals that also propagate the uncertainty of model estimation included zero for the NRI at every threshold. These analyses therefore indicate the direction of the difference but do not establish its magnitude (see Discussion Section).

### 3.5. Bootstrap Internal Validation

Both protocols showed excellent diagnostic performance across all steatosis thresholds (Table 4). Bootstrap internal validation (2000 resamples) confirmed negligible optimism (<0.001), and the optimism-corrected C-indices were 0.92, 0.92, and 0.90 for conventional UGAP and 0.92, 0.91, and 0.89 for WFUMB-based UGAP at the ≥S1, ≥S2, and S3 thresholds, respectively, identical to the apparent AUC values.

### 3.6. Factors Affecting Inter-Protocol Difference

Univariate analysis identified several factors associated with inter-protocol difference: age (ρ = −0.12; *p* < 0.01), etiology (MASLD vs. others; *p* < 0.01), BMI (ρ = 0.36; *p* < 0.01), skin-to-capsule distance (SCD) (ρ = 0.29; *p* < 0.01), aspartate aminotransferase (ρ = 0.10; *p* = 0.02), alanine aminotransferase (ρ = 0.19; *p* < 0.01), and MRI-PDFF (ρ = 0.30; *p* < 0.01). Sex, total bilirubin, gamma-glutamyl transpeptidase, platelet count and ALBI score showed no significant associations (Table 5). FIB-4 was weakly and inversely associated with the difference (ρ = −0.09; *p* = 0.04), whereas MRE (ρ = 0.05; *p* = 0.28) and 2D-SWE (ρ = 0.07; *p* = 0.08) were not (Table 5).

In multiple linear regression, BMI emerged as the strongest independent predictor (standardized β = 0.27; *p* < 0.01), followed by MRI-PDFF (β = 0.14; *p* < 0.01). SCD was not entered in the primary model because it was closely correlated with BMI (ρ = 0.81), and BMI was the more clinically accessible of the two. After adjusting for BMI, the apparent associations of etiology with inter-protocol difference were no longer significant. The multivariate model yielded an apparent R^2^ of 0.14, with an optimism-corrected R^2^ of 0.12 (bootstrap, 2000 resamples), indicating minimal overfitting. FIB-4 was not an independent predictor (β = −0.02; *p* = 0.68). In the sensitivity analysis with SCD entered in place of BMI and fitted on the same patients, SCD was an independent predictor of similar magnitude (standardized β = 0.22, 95% CI 0.13–0.31; *p* < 0.001), with MRI-PDFF also significant (β = 0.19; *p* < 0.001) and a slightly lower model R^2^ of 0.12 (optimism-corrected 0.10). When both variables were entered together, BMI retained significance (β = 0.27; *p* < 0.001), and SCD did not (β = 0.01; *p* = 0.95), with acceptable collinearity (variance inflation factors: 4.1 and 4.0). Because the two are strongly collinear, these coefficients cannot be interpreted as independent contributions; what they show is that either variable alone accounts for the association and that neither adds to the other (Table 5, footnote). Adding center as a fixed effect left the BMI and MRI-PDFF estimates essentially unchanged (β = 0.28 and 0.15, respectively; both *p* < 0.01); the joint contribution of center was modest but significant (F-test *p* = 0.02; R^2^ = 0.15).

### 3.7. Stratified Analysis by Skin-to-Capsule Distance

Diagnostic performance was compared within SCD subgroups (Supplementary Table S1). Correlation with MRI-PDFF favored the conventional protocol in the three larger strata (ρ = 0.759 vs. 0.733 for SCD < 20 mm, *p* = 0.031; 0.788 vs. 0.755 for SCD ≥ 20 mm, *p* = 0.041; 0.813 vs. 0.790 for SCD < 25 mm, *p* = 0.006) but not in the SCD ≥ 25 mm subgroup (0.645 vs. 0.584; *p* = 0.353; *n* = 51). In contrast, AUCs did not differ significantly between protocols at any threshold in any stratum (*p* = 0.14–0.77), although values were numerically higher for or equal to conventional UGAP throughout. SCD was not recorded in nine patients, who are absent from these strata. In the SCD ≥ 25 mm subgroup, only four patients had S0 steatosis, so both protocols separated ≥S1 steatosis completely and the DeLong test could not be computed.

### 3.8. Stratified Analysis by Fibrosis Stage

Diagnostic performance was compared within MRE-based fibrosis strata (Supplementary Table S3). Correlation with log MRI-PDFF was preserved in F0–1 (ρ = 0.843 vs. 0.825; *p* = 0.029) and F2–3 (ρ = 0.829 vs. 0.788; *p* = 0.012) and fell to a similar degree for both protocols in F4 (ρ = 0.521 vs. 0.508; *p* = 0.858; *n* = 30). AUCs did not differ significantly between protocols at any threshold in any stratum (*p* = 0.08–0.76). In F4, the median MRI-PDFF was 5.8%; no patient had S3 steatosis, and only four had S2 steatosis, so the AUC at the S3 threshold could not be estimated, and the ≥S2 estimate, based on four cases, is not reported.

After adjustment for log MRI-PDFF, BMI and SCD, MRE was not independently associated with the attenuation coefficient under either protocol (standardized β = 0.028, *p* = 0.26 for conventional UGAP; β = 0.033, *p* = 0.22 for WFUMB-based UGAP) (Supplementary Table S4). The difference between protocols was unrelated to MRE (ρ = 0.05; *p* = 0.28) and did not differ across strata (Kruskal–Wallis *p* = 0.33), with a median difference of 0.02 dB/cm/MHz in all three. The results were similar using 2D-SWE or FIB-4, although the difference in correlation was larger at higher 2D-SWE values (Supplementary Table S3, Panel B).

## 4. Discussion

To our knowledge, this is the first multicenter study evaluating WFUMB-recommended ROI settings on UGAP. The principal finding is that neither configuration showed a clinically meaningful advantage: AUCs did not differ significantly at any threshold, and the Obuchowski index differed by only 0.01. The correlation with MRI-PDFF (Δρ = 0.02) and the Obuchowski index favored the conventional configuration significantly, but with 565 patients, differences of this size are detectable without being clinically meaningful. The direction was consistent across metrics and across subgroups defined by body habitus and fibrosis, but not across centers, where the difference in correlation favored the conventional protocol at the largest center and the WFUMB-based protocol at the smallest. These results compare two ROI configurations within archived acquisitions and are not a head-to-head comparison of two prospectively acquired protocols; the interpretation that follows is framed around this asymmetry.

### 4.1. Acquisition Asymmetry as the Principal Alternative Explanation

The comparison was structurally asymmetric, and this is the simplest explanation for the small difference we found. Conventional measurements were made in real time, so the operator could adjust the probe, choose the respiratory phase, and reject poor frames while the automated fine-adjustment algorithm ran; WFUMB-based values were reconstructed from 5 to 10 archived frames without any of these corrections. Spatial sampling also differed because hepatic fat is unevenly distributed, and the two depth ranges do not overlap. Consequently, this study does not isolate the effect of ROI depth and length, and we cannot determine how much of the observed difference is attributable to the ROI configuration itself rather than to acquisition conditions. Our findings should therefore not be read as evidence that the WFUMB configuration is inferior, or that manufacturer-optimized settings are preferable in practice; they indicate only that, on this platform and under these conditions, the WFUMB configuration did not improve on the manufacturer’s default.

### 4.2. Statistical Significance Versus Clinical Relevance

The AUCs, the metrics most directly relevant to detecting steatosis at each threshold, did not differ. The correlation coefficient and Obuchowski index differed by 0.02 and 0.01, magnitudes well within the measurement variability reported for attenuation-based techniques [14,22], and a difference of 0.01 in ordinal discrimination is unlikely to change an individual patient’s steatosis grade. The IDI and category-free NRI, prespecified as secondary supportive analyses, were positive at all thresholds, but neither their magnitude nor their nominal significance should be read as clinical importance. Category-free NRI counts any shift in the predicted direction regardless of size, so it inflates small consistent differences, and its asymptotic test is anti-conservative [21]. In our data this is explicit: bootstrap confidence intervals for the NRI include zero at every threshold despite nominally significant asymptotic *p*-values. The IDI, which is less susceptible to this inflation, did not reach significance at the ≥S1 threshold at all—precisely the threshold at which the two AUCs were closest—and reached only 0.035 and 0.042 at the higher thresholds. We therefore regard these metrics as indicating the direction, and nothing more. A further caution applies to the whole comparison: we did not prespecify an equivalence margin and did not test for equivalence, so the absence of significant differences in AUC should be read as a failure to demonstrate an advantage for either configuration rather than as proof that the two are interchangeable. A difference too small to matter in an individual patient, yet systematic across a population, cannot be excluded.

### 4.3. Hepatic Fibrosis

Ultrasound attenuation cannot separate signal loss caused by fat from that caused by fibrosis. After adjustment for log MRI-PDFF, BMI and SCD, MRE was not independently associated with attenuation under either protocol, the inter-protocol difference was unrelated to MRE, and correlation with MRI-PDFF fell in F4 to a similar degree for both. Whether this reflects fibrosis itself is uncertain: the F4 stratum was small (*n* = 30), contained no patient with S3 steatosis and had a compressed MRI-PDFF range, so restriction of range is an equally plausible explanation. What can be said is that whatever degraded fat quantification in cirrhosis did so symmetrically, and conclusions in this group remain tentative.

### 4.4. Possible Mechanisms: Hypotheses Not Tested in This Study

Three physical mechanisms could contribute to the small advantage of the deeper configuration; none was tested in the present study, and they are offered as hypotheses derived from previous work rather than as explanations supported by our data. First, attenuation bias is minimized when the transmit focal zone lies beyond the ROI [23], and better MRI-PDFF agreement has been reported at deeper settings [14,24]; the 4–8 cm window may align more closely with the elevational focus of the C1-6 transducer. Second, shallow ROI placement is susceptible to reverberation clutter and phase aberration from subcutaneous tissue [25,26,27], and, consistent with but not proof of this mechanism, the inter-protocol difference increased with BMI (β = 0.27) and SCD. Third, UGAP employs phantom-based calibration optimized for the 4 cm/4 cm geometry [28], around which the focal zone, time-gain compensation and noise filters are built; repositioning the ROI moves the measurement outside this geometry. A protocol that is sound in principle may therefore underperform on a given platform for reasons unrelated to the principle. Dedicated phantom and in vivo experiments would be needed to confirm any of these mechanisms.

The ultrasound and MRI sampling volumes also differ, and exact correspondence cannot be guaranteed; this limits the correlation achievable by either protocol but affects both equally, so it is not a source of differential bias.

### 4.5. Body Habitus

BMI was the strongest independent predictor of the inter-protocol difference, and SCD was an independent predictor of similar magnitude when substituted for it (β = 0.22 vs. 0.27). The two were highly collinear (ρ = 0.81) and neither added to the other, which is consistent with both reflecting a common aspect of body habitus—plausibly the thickness of the tissue layer through which the shallow window, but not the deep one, must sample—although these data cannot establish which physical quantity is responsible. BMI was retained in the primary model because it explained marginally more of the variance and is recorded routinely; this is a pragmatic choice rather than evidence that BMI is the better physical descriptor, and with collinearity of this degree the two models should not be read as competing. The center-level pattern was consistent with this: the inter-protocol difference was largest at the two centers with the highest median BMI, and a modest residual center effect remained after adjustment, which may reflect differences in operator technique that a retrospective design cannot resolve. Our cohort, however, had short SCD (median 19.0 mm), with only three patients ≥ 30 mm, so the comparison is essentially uninformative for the body habitus common in Western populations with obesity. The question is of practical relevance: a start depth fixed 2 cm below the capsule places the window at different absolute depths in thin and obese patients, whereas one fixed 4 cm from the skin may fall too close to the capsule when SCD is large. Whether the optimal depth should vary with body habitus rather than being universally fixed is a hypothesis our data raise but cannot answer, and validation in populations with greater obesity should be a priority.

### 4.6. Platform Specificity and the Broader Context

The optimal ROI configuration may be device-specific. Ferraioli et al. [29] reported that a movable ROI outperformed the fixed setting with the Fujifilm iATT system, contrasting with our findings. This discrepancy may reflect calibration strategy: UGAP uses phantom-based calibration aligned to 4 cm/4 cm, whereas iATT uses a reference table for depth adjustment, and its fixed ROI (35–75 mm from the transducer) may lie too close to the capsule when subcutaneous tissue is thick. Since attenuation-based techniques perform well on other platforms under their own optimized settings [12,22], the principle appears robust while the best ROI configuration may differ between systems. Our results demonstrate platform-specific behavior on one system; they do not show that manufacturer-specific calibration is generally preferable to a harmonized protocol across ultrasound systems, and cross-study comparisons of this kind are indirect and hypothesis-generating.

Operator reproducibility, a primary goal of standardized protocols, could not be addressed by our retrospective design. Interobserver agreement is excellent on other platforms [22] but depends on measurement parameters, including ROI depth [14]. Whether the reproducibility of the WFUMB configuration on UGAP differs from the manufacturer’s default remains unknown and should be evaluated prospectively.

The WFUMB guidance is advisory rather than binding and acknowledges that device-specific optimization may differ [13]. Standardization across vendors is limited by differences in transducer bandwidth, acoustic power, pulse coding and proprietary algorithms [30,31], which yield platform-specific cutoffs [29], and by organizational as much as technical barriers [32]. Our results are therefore not a challenge to the WFUMB recommendations, but a reminder that clinical judgment about one’s own system and patients is still required when guidance is applied to a specific platform [33].

### 4.7. Strengths and Limitations

One methodological advantage of this study is the centralized, blinded reanalysis of WFUMB-based UGAP measurements from archived raw data: because both configurations were derived from identical acquisitions, probe position, respiratory phase and tissue compression were constant between them and cannot explain the difference. The same design feature is the source of the acquisition asymmetry discussed above since only the conventional measurements benefited from real-time selection among frames. The large multicenter cohort provided robust statistical power, and bootstrap internal validation confirmed negligible optimism for all C-indices.

This study has several limitations. First, and most importantly, the comparison was asymmetric: the WFUMB-based values were derived retrospectively from stored data without real-time operator optimization, whereas the conventional values were acquired prospectively with such optimization. This study therefore does not represent a balanced head-to-head comparison of two prospectively acquired protocols, and the WFUMB configuration was not applied under the conditions for which it was designed. Second, selection bias cannot be excluded. Raw data were stored at the discretion of each center, and 430 of 1010 patients (43%) were excluded because no raw data had been stored, with a further 15 excluded for insufficient quality or data loss. Although availability depended on workflow rather than on patient characteristics, the analysis cohort had a lower median MRI-PDFF (7.4% vs. 9.3%) and greater subcutaneous fat thickness (19.8 vs. 17.6 mm) than excluded patients (Supplementary Table S2, Panel A). Because the inter-protocol difference increased with both body habitus and attenuation values (proportional bias), exclusion of patients with higher steatosis burden may have modestly underestimated the inter-protocol difference; this would, if anything, favor the WFUMB configuration and does not account for the small advantage of the conventional configuration. Exclusion was, however, concentrated at the two centers that did not store raw data at all (356 of 445 exclusions), whose case mix largely explains these differences; within the four contributing centers, 78–92% of patients were analyzable, and included and excluded patients did not differ significantly in any measured characteristic (Supplementary Table S2, Panel B; Supplementary Table S5). This analysis can exclude selection on the characteristics we recorded, but not on unmeasured ones. The 15 patients excluded for poor data quality were too few to characterize separately. The inter-protocol difference nevertheless differed across centers; this heterogeneity tracked center-level body habitus, and a modest residual center effect remained after adjustment, so operator- or center-related factors cannot be excluded. The 100% measurement success rate likewise applies only to the selected cohort with analyzable raw data. Third, all examinations were performed on a single vendor platform (GE LOGIQ E10) at Japanese centers, and two co-authors are employees of GE HealthCare; our findings may not be generalizable to other systems. Fourth, patients with SCD ≥ 30 mm, as commonly encountered in Western cohorts, were underrepresented (*n* = 3; maximum 31 mm), and our findings cannot be extrapolated to such populations. Fifth, histological confirmation was not implemented; although MRI-PDFF is a well-validated reference standard, the use of a single 20 × 20 × 20 mm^3^ ROI in segment VII or VIII may not fully reflect the spatial heterogeneity of hepatic fat deposition [34,35]. Sixth, inter- and intra-operator variability was not formally assessed for either protocol, and all sonographers had extensive experience (>1000 examinations); diagnostic performance may differ with less experienced operators. Seventh, our cohort predominantly comprised patients with MASLD, which may have limited the applicability to other etiologies. Eighth, serum ferritin was not recorded, and R2* maps were not stored, so hepatic iron overload could not be excluded; because both protocols derive from the same acquisitions, iron would be expected to affect them equally. Finally, the mechanistic explanations offered above were not tested directly.

The definitive comparison would be a prospective paired study in which both configurations are acquired in real time in the same patients, each with its own operator optimization, with assessment of inter- and intra-operator reproducibility across a wide range of body habitus. Such a design would establish whether the small differences observed here arise from the ROI configuration itself or from acquisition asymmetry.

## 5. Conclusions

In this single-vendor multicenter study, neither the manufacturer-optimized (4 cm/4 cm) nor the WFUMB-recommended (2 cm/3 cm) ROI configuration showed a clinically meaningful advantage for hepatic steatosis against MRI-PDFF: AUCs did not differ significantly at any threshold, and overall ordinal discrimination differed by 0.01. Equivalence was not formally tested, so this is an absence of demonstrable advantage rather than a demonstration of equivalence. Small numerical differences favored the manufacturer-optimized configuration in the pooled analysis, although the direction was not uniform across centers; because the WFUMB-based values were reconstructed retrospectively without real-time optimization, acquisition asymmetry may account for much of this difference, and our results do not show that the WFUMB configuration is inferior. The findings come from a single platform and a population with relatively short skin-to-capsule distances; they are hypothesis-generating and indicate that the optimal ROI configuration may be platform-specific. A prospective paired study with real-time acquisition of both configurations is needed before either can be recommended over the other.

## Figures and Tables

**Table 1:** Clinical features and laboratory data of the study cohort.

Characteristics	*n* = 565	
Age (years)	64	[52–72]
Sex (male, %)	302	(53.5)
Etiology (HBV/HCV/MASLD/ALD/Others)	89/67/293/37/79	
SCD (mm)	19.0	[16.0–21.0]
BMI (kg/m^2^)	25.4	[22.9–28.2]
T.Bil (mg/dL)	0.8	[0.6–1.0]
AST (U/L)	31	[22.0–49.0]
ALT (U/L)	33	[20.0–58.0]
GGT (U/L)	45	[25.0–83.0]
ALBI score	−2.99	[−3.16 to −2.74]
Plt (×10^4^/mm^3^)	20.6	[15.9–24.9]
FIB-4 score	1.79	[1.12–2.70]
MRE at 60 Hz (kPa)	2.84	[2.25–3.90]
MRI-PDFF (%)	7.4	[3.1–14.9]

Note—The values represent the median [25–75th percentile] or number of patients. Abbreviations: HBV, hepatitis B virus; HCV, hepatitis C virus; MASLD, metabolic dysfunction-associated steatotic liver disease; SCD, skin-to-capsule distance; BMI, body mass index; T.Bil, total bilirubin; AST, aspartate aminotransferase; ALT, alanine aminotransferase; GGT, gamma-glutamyl transpeptidase; ALBI, albumin–bilirubin; Plt, platelet; FIB-4, Fibrosis-4 index; PDFF, proton density fat fraction. The FIB-4 index was calculated as (age × AST)/(platelet count [10^9^/L] × √ALT). Magnetic resonance elastography was available in 561 of the 565 patients. Skin-to-capsule distance was not recorded in 9 patients.

**Table 2:** Diagnostic performance of conventional and WFUMB-based UGAP for detecting hepatic steatosis.

	≥S1 (*n* = 347)		≥S2 (*n* = 201)		S3 (*n* = 113)	
	Conventional	WFUMB-Based	Conventional	WFUMB-Based	Conventional	WFUMB-Based
AUC (95%CI)	0.92 (0.90–0.94)	0.92 (0.89–0.94)	0.92 (0.90–0.94)	0.91 (0.88–0.93)	0.90 (0.87–0.93)	0.89 (0.86–0.92)
Cutoff	0.69	0.65	0.72	0.70	0.77	0.74
Sensitivity	0.79 (0.75–0.83)	0.86 (0.82–0.89)	0.88 (0.82–0.91)	0.84 (0.78–0.88)	0.80 (0.71–0.86)	0.80 (0.71–0.86)
Specificity	0.91 (0.87–0.94)	0.85 (0.80–0.89)	0.82 (0.77–0.85)	0.81 (0.77–0.85)	0.84 (0.80–0.87)	0.84 (0.80–0.87)
PPV	0.94 (0.90–0.96)	0.90 (0.86–0.93)	0.72 (0.66–0.78)	0.71 (0.65–0.77)	0.55 (0.48–0.63)	0.55 (0.47–0.62)
NPV	0.73 (0.68–0.78)	0.79 (0.74–0.84)	0.92 (0.89–0.95)	0.90 (0.86–0.93)	0.94 (0.92–0.96)	0.94 (0.92–0.96)
LR+	9.09 (6.28–15.25)	5.69 (4.31–8.24)	4.76 (3.86–6.01)	4.47 (3.62–5.71)	4.93 (3.92–6.35)	4.86 (3.93–6.22)
LR−	0.23 (0.18–0.27)	0.16 (0.12–0.21)	0.15 (0.10–0.21)	0.20 (0.14–0.27)	0.24 (0.16–0.34)	0.24 (0.16–0.34)

Note—Abbreviations: WFUMB, World Federation for Ultrasound in Medicine and Biology; AUC, area under the receiver operating characteristic curve; CI, confidence interval; PPV, positive predictive value; NPV, negative predictive value; LR+, positive likelihood ratio; LR−, negative likelihood ratio; UGAP, ultrasound-guided attenuation parameter.

**Table 3:** Comparison of discrimination metrics between conventional and WFUMB-based UGAP protocols.

Metric	Conventional	WFUMB-Based	*p*-Value
Obuchowski Index	0.92 (0.90–0.93)	0.91 (0.89–0.92)	0.04
IDI (≥S1)	0.021 (−0.002 to 0.044)	reference	0.07
IDI (≥S2)	0.035 (0.014 to 0.056)	reference	<0.01
IDI (S3)	0.042 (0.015 to 0.068)	reference	<0.01
NRI (≥S1)	0.24 (0.07 to 0.41)	reference	<0.01
NRI (≥S2)	0.38 (0.21 to 0.55)	reference	<0.01
NRI (S3)	0.53 (0.33 to 0.73)	reference	<0.01

Note—Abbreviations: WFUMB, World Federation for Ultrasound in Medicine and Biology; IDI, integrated discrimination improvement; NRI, category-free net reclassification improvement. The Obuchowski index was the primary metric of overall discrimination; it was computed as the weighted average of the pairwise areas under the curve for all pairs of steatosis grades, weighting each pair by the absolute difference in grade and by the number of discordant pairs. IDI and category-free NRI, with WFUMB-based UGAP as the reference, were prespecified secondary supportive analyses and are given with asymptotic 95% confidence intervals and *p*-values; IDI is reported to three decimal places because the estimates lie close to zero. Bootstrap percentile intervals (2000 resamples), which additionally propagate the uncertainty of re-estimating the models, are considerably wider: for the IDI, −0.015 to 0.057, 0.002 to 0.069 and 0.004 to 0.079, and for the NRI, −0.29 to 0.75, −0.08 to 0.78 and 0.00 to 0.88 at the ≥S1, ≥S2 and S3 thresholds, so the interval for the NRI includes zero at every threshold. The IDI did not reach significance at the ≥S1 threshold, which is also the threshold at which the two areas under the curve were closest. These analyses therefore indicate the direction, not the clinical importance, of the difference.

**Table 4:** Bootstrap internal validation of diagnostic performance: optimism-corrected C-indices for conventional and WFUMB-based UGAP (with WFUMB-based UGAP as the comparator).

	C-Index (95% CI)	*p*-Value
Conventional UGAP (≥S1)	0.92 (0.90–0.94)	0.23
WFUMB-based UGAP (≥S1)	0.92 (0.89–0.94)	reference
Conventional UGAP (≥S2)	0.92 (0.90–0.94)	0.07
WFUMB-based UGAP (≥S2)	0.91 (0.88–0.93)	reference
Conventional UGAP (S3)	0.90 (0.87–0.93)	0.14
WFUMB-based UGAP (S3)	0.89 (0.86–0.92)	reference

Note—C-indices were calculated using bootstrap resampling (2000 iterations). Optimism was negligible (<0.001) for all models. *p*-values were obtained by DeLong test comparing each protocol against WFUMB-based UGAP. Abbreviations: UGAP, ultrasound-guided attenuation parameter; WFUMB, World Federation for Ultrasound in Medicine and Biology; C-index, concordance index.

**Table 5:** Factors associated with inter-protocol differences in UGAP values.

	Univariate Analysis		Multivariate Analysis		
Variable	*ρ*	*p*-Value	*β*	(95%CI)	*p*-Value
Age	−0.12	<0.01	0.03	(−0.06 to 0.13)	0.48
Sex (male/female)	—	0.71			
Etiology (MASLD/non-MASLD)	—	<0.01	0.01	(−0.09 to 0.10)	0.92
SCD	0.29	<0.01			
BMI	0.36	<0.01	0.27	(0.18–0.37)	<0.01
T.Bil	0.00	0.96			
AST	0.10	0.02	−0.06	(−0.21 to 0.10)	0.48
ALT	0.19	<0.01	0.05	(−0.11 to 0.20)	0.55
GGT	0.07	0.08			
ALBI score	−0.02	0.68			
Plt	0.03	0.48			
FIB-4 score	−0.09	0.04	−0.02	(−0.13 to 0.08)	0.68
MRE at 60 Hz (kPa)	0.05	0.28			
2D-SWE (kPa)	0.07	0.08			
MRI-PDFF	0.30	<0.01	0.14	(0.04 to 0.25)	<0.01

R^2^ = 0.14 for multivariate model (*n* = 556); *ρ*, Spearman correlation coefficient; *β*, standardized regression coefficient. The FIB-4 index, magnetic resonance elastography and 2D shear wave elastography were included as measures of hepatic fibrosis. None were independently associated with the inter-protocol difference; BMI and MRI-PDFF were the only independent predictors. Abbreviations: MASLD, metabolic dysfunction-associated steatotic liver disease; SCD, skin-to-capsule distance; BMI, body mass index; T.Bil, total bilirubin; AST, aspartate aminotransferase; ALT, alanine aminotransferase; GGT, gamma-glutamyl transpeptidase; ALBI, albumin–bilirubin; Plt, platelet; FIB-4, Fibrosis-4 index; PDFF, proton density fat fraction. Variables with *p* < 0.05 in univariate analysis were considered in multivariate analysis. SCD was not entered because it was closely correlated with BMI (ρ = 0.81), and BMI was the more clinically accessible of the two; all variance inflation factors in the final model were below 5 (maximum 4.0). Sensitivity analyses on the same complete-case set (*n* = 556): With SCD entered in place of BMI: SCD β = 0.22 (95% CI 0.13 to 0.31), *p* < 0.001; MRI-PDFF, β = 0.19 (0.08 to 0.29), *p* < 0.001; R^2^ = 0.12 (optimism-corrected 0.10). With BMI and SCD entered together, BMI retained significance (β = 0.27, 0.11 to 0.43; *p* < 0.001), and SCD did not (β = 0.01, −0.15 to 0.16; *p* = 0.95), with variance inflation factors of 4.1 and 4.0. Adding center as a fixed effect did not materially change the estimates for BMI (β = 0.28) or MRI-PDFF (β = 0.15) (Supplementary Table S5). Skin-to-capsule distance was not recorded in 9 patients.

## Data Availability

The data that support the findings of this study are available from the corresponding author upon reasonable request.

## Supplementary Materials

The following supporting information can be downloaded at https://www.mdpi.com/article/10.3390/jcm15218373/s1. Table S1: Stratified analysis of diagnostic performance by skin-to-capsule distance; Table S2: Comparison of clinical characteristics between the analysis cohort and the excluded patients, overall (Panel A) and within the centers that contributed analyzable data (Panel B); Table S3: Stratified analysis of diagnostic performance by hepatic fibrosis stage; Table S4: Effect of hepatic fibrosis on attenuation after adjustment for MRI-PDFF and association of the inter-protocol difference with fibrosis; Table S5: Center-specific inclusion numbers, recruitment period and diagnostic performance.

## Abbreviations

ALBI, albumin–bilirubin; ALT, alanine aminotransferase; AST, aspartate aminotransferase; AUC, area under the receiver operating characteristic curve; BMI, body mass index; CI, confidence interval; EFSUMB, European Federation of Societies for Ultrasound in Medicine and Biology; FIB-4, Fibrosis-4 index; HBV, hepatitis B virus; HCV, hepatitis C virus; ICC, intraclass correlation coefficient; IDI, integrated discrimination improvement; MASLD, metabolic dysfunction-associated steatotic liver disease; MRE, magnetic resonance elastography; MRI-PDFF, magnetic resonance imaging-derived proton density fat fraction; NRI, net reclassification improvement; PDFF, proton density fat fraction; ROC, receiver operating characteristic; ROI, region of interest; SCD, skin-to-capsule distance; STROBE, Strengthening the Reporting of Observational Studies in Epidemiology; SWE, shear wave elastography; UGAP, ultrasound-guided attenuation parameter; WFUMB, World Federation for Ultrasound in Medicine and Biology.
