# Supplementary material for: WFUMB-Recommended Versus Manufacturer-Optimized Protocols for Ultrasound-Guided Attenuation Parameter in Hepatic Steatosis Assessment: A Retrospective Single-Vendor Multicenter Study
Source: J Clin Med. 2026 Oct 27;15(21):8373. doi: 10.3390/jcm15218373 (PMC13764524; doi:10.3390/jcm15218373)
Supplement: Supplementary file 1 [file jcm-15-08373-s001.zip › Supplementary_Table_S5.pdf]

Supplementary Table S5. Center-specific inclusion numbers, recruitment period and diagnostic performance.

Center	Recruitment period	Registered, n	Analyzable raw data, n (%)	Steatosis ≥S1, n (%)	Median SCD (mm)	Median BMI (kg/m²)	ρ with log MRI-PDFF, Conventional	ρ with log MRI-PDFF, WFUMB-based	*p* (Steiger)	AUC ≥S1, Conventional (95% CI)	AUC ≥S1, WFUMB-based (95% CI)	*p* (DeLong)	Median inter-protocol difference, dB/cm/MHz [IQR]
Center 1	Feb 2020–Jan 2021	310	286 (92.3)	153 (53.5)	17.0	24.9	0.820	0.781	<0.001	0.924 (0.891–0.953)	0.901 (0.864–0.935)	0.01	0.010 [−0.010 to 0.030]
Center 2	Mar 2020–Feb 2021	196	153 (78.1)	121 (79.1)	20.0	27.5	0.823	0.794	0.074	0.950 (0.914–0.979)	0.955 (0.919–0.984)	0.74	0.020 [−0.010 to 0.040]
Center 3	Feb 2020–Dec 2020	106	91 (85.8)	45 (49.5)	18.0	24.2	0.740	0.715	0.352	0.846 (0.757–0.917)	0.855 (0.775–0.924)	0.63	0.020 [−0.010 to 0.050]
Center 4	Jul 2020–Dec 2020	42	35 (83.3)	28 (80.0)	20.0	28.3	0.781	0.870	0.032	0.918 (0.780–1.000)	0.962 (0.885–1.000)	0.20	0.040 [0.010 to 0.070]
Center 5	—	49	0 (0)	—	—	—	—	—	—	—	—	—	—
Center 6	—	307	0 (0)	—	—	—	—	—	—	—	—	—	—
Total	Feb 2020–Feb 2021	1,010	565 (55.9)	347 (61.4)	19.0	25.4	0.830	0.805	<0.001	0.923 (0.900–0.944)	0.915 (0.890–0.939)	0.23	0.020 [−0.010 to 0.040]

Note.– Centers are numbered in descending order of the number of analyzable examinations and are not identified. Raw data were stored at the discretion of each center when storage capacity and examination time permitted; at Centers 5 and 6 raw data were not stored during the original examinations, so none of their patients could be reanalyzed, and these two centers accounted for 356 of the 445 exclusions (80%). Recruitment period is the range of ultrasound examination dates. Spearman ρ with log MRI-PDFF was compared between protocols within each center by Steiger's Z-test for dependent correlations, and AUCs for ≥S1 steatosis by the DeLong test; AUC confidence intervals are bootstrap percentile intervals (2,000 resamples). AUCs are given to three decimal places because the between-protocol differences are smaller than 0.01 at some centers. The inter-protocol difference (Conventional − WFUMB-based) differed across the four contributing centers (Kruskal–Wallis H = 19.3, $p < 0.001$) and was largest at the two centers with the highest median BMI. In the multivariable model of the inter-protocol difference, adding center as a fixed effect did not materially change the estimates for BMI (standardized $\beta = 0.28$, $p < 0.001$) or MRI-PDFF ($\beta = 0.15$, $p = 0.008$); the joint contribution of center was modest but significant (F-test $p = 0.02$; $R^2 = 0.15$ versus 0.14 without center). Estimates for Center 4 (n = 35) should be interpreted with caution.

Abbreviations: AUC, area under the receiver operating characteristic curve; BMI, body mass index; CI, confidence interval; IQR, interquartile range; MRI-PDFF, magnetic resonance imaging-derived proton density fat fraction; SCD, skin-to-capsule distance; UGAP, ultrasound-guided attenuation parameter; WFUMB, World Federation for Ultrasound in Medicine and Biology.
