# Supplementary material for: WFUMB-Recommended Versus Manufacturer-Optimized Protocols for Ultrasound-Guided Attenuation Parameter in Hepatic Steatosis Assessment: A Retrospective Single-Vendor Multicenter Study
Source: J Clin Med. 2026 Oct 27;15(21):8373. doi: 10.3390/jcm15218373 (PMC13764524; doi:10.3390/jcm15218373)
Supplement: Supplementary file 1 [file jcm-15-08373-s001.zip › Supplementary_Table_S1.pdf]

Supplementary Table S1. Stratified analysis of diagnostic performance by skin-to-capsule distance.

	**SCD < 20 mm**	**SCD ≥ 20 mm**	**SCD < 25 mm***	**SCD ≥ 25 mm***
**n**	351	205	505	51
**Steatosis grade**				
S0, n (%)	189 (53.8)	28 (13.7)	213 (42.2)	4 (7.8)
S1, n (%)	79 (22.5)	62 (30.2)	133 (26.3)	8 (15.7)
S2, n (%)	44 (12.5)	42 (20.5)	74 (14.7)	12 (23.5)
S3, n (%)	39 (11.1)	73 (35.6)	85 (16.8)	27 (52.9)
**Spearman ρ with MRI-PDFF**				
Conventional UGAP	0.759	0.788	0.813	0.645
WFUMB-based UGAP	0.733	0.755	0.790	0.584
p (Steiger's Z)	0.031	0.041	0.006	0.353
**AUC ≥S1 (95% CI)**				
Conventional	0.89 (0.86–0.92)	0.94 (0.90–0.97)	0.91 (0.89–0.93)	1.00 (1.00–1.00)
WFUMB-based	0.89 (0.85–0.92)	0.93 (0.88–0.97)	0.90 (0.88–0.93)	1.00 (1.00–1.00)
p (DeLong test)	0.77	0.48	0.32	NC
**AUC ≥S2 (95% CI)**				
Conventional	0.92 (0.89–0.95)	0.90 (0.85–0.94)	0.92 (0.89–0.94)	0.89 (0.79–0.96)
WFUMB-based	0.91 (0.87–0.94)	0.89 (0.84–0.93)	0.91 (0.88–0.93)	0.84 (0.72–0.93)

p (DeLong test)	0.14	0.39	0.14	0.23
**AUC S3 (95% CI)**				
Conventional	0.90 (0.86–0.94)	0.87 (0.82–0.92)	0.90 (0.87–0.93)	0.79 (0.66–0.89)
WFUMB-based	0.90 (0.86–0.93)	0.85 (0.80–0.90)	0.90 (0.86–0.93)	0.76 (0.62–0.87)
p (DeLong test)	0.59	0.17	0.30	0.49

Note.– Data are presented as n (%) for steatosis grade distribution. AUC values are shown with 95% bootstrap confidence intervals (2,000 resamples). Between-protocol comparisons used the DeLong test for AUCs and Steiger's Z-test for dependent correlations. In the SCD ≥ 25 mm subgroup only four patients had S0 steatosis, so both protocols separated ≥S1 steatosis completely (AUC 1.00) and the DeLong test could not be computed (NC). *SCD ≥ 25 mm subgroup results should be interpreted with caution given the small sample size (n = 51). Skin-to-capsule distance was not recorded in 9 patients, who are therefore absent from all strata; the strata accordingly comprise 556 of the 565 analyzed patients. Abbreviations: AUC, area under the receiver operating characteristic curve; CI, confidence interval; MRI-PDFF, magnetic resonance imaging-derived proton density fat fraction; SCD, skin-to-capsule distance; UGAP, ultrasound-guided attenuation parameter; WFUMB, World Federation for Ultrasound in Medicine and Biology.
