# Supplementary material for: WFUMB-Recommended Versus Manufacturer-Optimized Protocols for Ultrasound-Guided Attenuation Parameter in Hepatic Steatosis Assessment: A Retrospective Single-Vendor Multicenter Study
Source: J Clin Med. 2026 Oct 27;15(21):8373. doi: 10.3390/jcm15218373 (PMC13764524; doi:10.3390/jcm15218373)
Supplement: Supplementary file 1 [file jcm-15-08373-s001.zip › Supplementary_Table_S2.pdf]

Supplementary Table S2. Comparison of clinical characteristics between the analysis cohort and the excluded patients, overall (Panel A) and within the centers that contributed analyzable data (Panel B).

Panel A. Analysis cohort versus all excluded patients.

Variable	Analysis cohort (n = 565)	Excluded patients (n = 445)	p-value
Age (years)	64 [52–72]	64 [52–72]	0.732
Male, n (%)	302 (53.5)	241 (54.2)	0.873
BMI (kg/m²)	25.4 [22.9–28.2]	26.0 [23.3–29.4]	0.077
Subcutaneous fat thickness (mm)*	19.8 [16.0–22.1]	17.6 [14.3–21.3]	<0.001
MRI-PDFF (%)	7.4 [3.1–14.9]	9.3 [5.8–16.8]	<0.001
**Steatosis grade**			
S0, n (%)	218 (38.6)	138 (31.0)	
S1, n (%)	146 (25.8)	135 (30.3)	
S2, n (%)	88 (15.6)	80 (18.0)	
S3, n (%)	113 (20.0)	92 (20.7)	

Note.– Continuous variables are presented as median [interquartile range]. Categorical variables are presented as n (%). p-values were calculated using the Mann-Whitney U test for continuous variables and the chi-square test for categorical variables; the distribution of steatosis grade did not differ between groups (p = 0.079). Of the 1,010 patients in the original cohort, 445 were excluded because raw data were not stored during the original examination (n = 430), were of insufficient quality for retrospective reanalysis (n = 13), or were inadvertently lost (n = 2). Of the 445 excluded patients, 356 were registered at the two centers that stored no raw data at all and 89 at the four centers that did (Supplementary Table S5). *Body habitus in this panel is the measurement recorded in the parent study, which is the only such measurement available for the excluded patients. The

skin-to-capsule distance used in the present analyses and reported in Table 1 was measured on the stored images at the time of the region-of-interest reanalysis and exists only for the analysis cohort; the two correlate moderately (Spearman ρ = 0.60), so this row should be read as a comparison of the parent-study measurement.

Abbreviations: BMI, body mass index; MRI-PDFF, magnetic resonance imaging-derived proton density fat fraction; SCD, skin-to-capsule distance.

Panel B. Analysis cohort versus patients excluded at the four centers that contributed analyzable data.

Variable	Analysis cohort (n = 563)	Excluded patients (n = 91)	p-value
Age (years)	64 [52–72]	65 [52–73]	0.852
Male, n (%)	301 (53.5)	51 (56.0)	0.730
BMI (kg/m²)	25.4 [22.9–28.3]	26.1 [23.4–29.8]	0.358
Subcutaneous fat thickness (mm)*	19.9 [16.0–22.1]	20.0 [16.0–23.1]	0.624
MRI-PDFF (%)	7.4 [3.1–14.9]	9.6 [4.8–15.6]	0.069
Steatosis grade			0.164
S0, n (%)	216 (38.4)	24 (26.4)	
S1, n (%)	146 (25.9)	30 (33.0)	
S2, n (%)	88 (15.6)	17 (18.7)	
S3, n (%)	113 (20.1)	20 (22.0)	

Note.– Two of the six participating centers did not store raw data during the original examinations, so none of their 356 registered patients could be reanalyzed (Supplementary Table S5). Panel B therefore restricts the comparison to the four centers that did store raw data, in which 654 patients were registered. Included and excluded patients within these centers did not differ significantly in any measured characteristic, indicating that the differences in Panel A reflect the case mix of the two non-contributing centers rather than selection at the level of individual patients. Panel B was constructed by linking the analysis dataset to the original registry; 2 of the 565 analyzed patients could not be matched and are counted in the excluded column (n = 91 rather than 89), which does not affect any comparison. Continuous variables are median [interquartile range]; p-values by Mann–Whitney U test for continuous variables and chi-square test for sex and steatosis grade. *Measurement as defined in Panel A.
