# Supplementary material for: WFUMB-Recommended Versus Manufacturer-Optimized Protocols for Ultrasound-Guided Attenuation Parameter in Hepatic Steatosis Assessment: A Retrospective Single-Vendor Multicenter Study
Source: J Clin Med. 2026 Oct 27;15(21):8373. doi: 10.3390/jcm15218373 (PMC13764524; doi:10.3390/jcm15218373)
Supplement: Supplementary file 1 [file jcm-15-08373-s001.zip › Supplementary_Table_S3.pdf]

Supplementary Table S3. Stratified analysis of diagnostic performance by hepatic fibrosis stage.

	F0–1 (n = 363)	F2–3 (n = 168)	F4 (n = 30)
**Steatosis grade, n (%)**			
S0	139 (38.3)	66 (39.3)	12 (40.0)
S1	91 (25.1)	40 (23.8)	14 (46.7)
S2	50 (13.8)	34 (20.2)	4 (13.3)
S3	83 (22.9)	28 (16.7)	0 (0.0)
Median MRI-PDFF (%)	7.7	7.2	5.8
Median MRE at 60 Hz (kPa)	2.40	4.30	7.75
Median SCD (mm)	18.0	19.0	17.0
Median BMI (kg/m²)	25.0	25.7	25.8
**Spearman ρ with log MRI-PDFF (95% CI)**			
Conventional UGAP	0.843 (0.808–0.872)	0.829 (0.772–0.874)	0.521 (0.176–0.752)
WFUMB-based UGAP	0.825 (0.786–0.857)	0.788 (0.719–0.842)	0.508 (0.159–0.744)
p (Steiger's Z)	0.029	0.012	0.858
**AUC ≥S1 (95% CI)**			
Conventional UGAP	0.93 (0.91–0.96)	0.94 (0.90–0.97)	0.73 (0.53–0.90)
WFUMB-based UGAP	0.93 (0.90–0.95)	0.93 (0.88–0.96)	0.71 (0.51–0.89)
p (DeLong test)	0.54	0.30	0.76
**AUC ≥S2 (95% CI)**			
Conventional UGAP	0.93 (0.90–0.95)	0.92 (0.87–0.96)	NC
WFUMB-based UGAP	0.92 (0.89–0.95)	0.90 (0.84–0.94)	NC
p (DeLong test)	0.52	0.08	NC
**AUC S3 (95% CI)**			
Conventional UGAP	0.91 (0.88–0.94)	0.88 (0.82–0.93)	NC
WFUMB-based UGAP	0.90 (0.87–0.93)	0.86 (0.79–0.92)	NC
p (DeLong test)	0.26	0.25	NC

Note.– Fibrosis stage was assigned from magnetic resonance elastography (MRE) at 60 Hz. Four patients without MRE data were excluded (n = 561). AUC values are shown with 95% bootstrap confidence intervals (2,000 resamples); between-protocol comparisons used the DeLong test. Spearman ρ values were compared using Steiger's Z-test for dependent correlations. In the F4 stratum no patient had S3 steatosis and only four had S2 steatosis (median MRI-PDFF 5.8%), so AUC at the ≥S2 and S3 thresholds could not be computed (NC); estimates in this stratum should be interpreted with caution given the small sample size (n = 30).

Panel B. Secondary stratification by 2D shear wave elastography and the FIB-4 index.

Stratification	Subgroup	n	ρ Conventional	ρ WFUMB-based	Difference	p
2D-SWE (kPa)	<7.0	307	0.841	0.832	+0.009	0.318
2D-SWE (kPa)	7.0–10.0	148	0.834	0.781	+0.053	<0.001
2D-SWE (kPa)	≥10.0	104	0.774	0.717	+0.058	0.015
FIB-4 index	<1.30	187	0.838	0.823	+0.015	0.222
FIB-4 index	1.30–2.67	232	0.845	0.807	+0.037	0.002
FIB-4 index	>2.67	146	0.710	0.681	+0.029	0.178

Note.– Spearman ρ with log MRI-PDFF. Difference = ρ (Conventional) − ρ (WFUMB-based); p by Steiger's Z-test. 2D-SWE was available in 559 patients and the FIB-4 index in 565. Because 2D-SWE is itself an ultrasound-based measurement and is influenced by body habitus (ρ with BMI = 0.21; ρ with SCD = 0.17, versus 0.17 and 0.12 for MRE), the MRE-based stratification in Panel A is regarded as the primary analysis.

Abbreviations: AUC, area under the receiver operating characteristic curve; BMI, body mass index; CI, confidence interval; FIB-4, Fibrosis-4 index; MRE, magnetic resonance elastography; MRI-PDFF, magnetic resonance imaging-derived proton density fat fraction; SCD, skin-to-capsule distance; SWE, shear wave elastography; UGAP, ultrasound-guided attenuation parameter; WFUMB, World Federation for Ultrasound in Medicine and Biology.
