# Supplementary material for: WFUMB-Recommended Versus Manufacturer-Optimized Protocols for Ultrasound-Guided Attenuation Parameter in Hepatic Steatosis Assessment: A Retrospective Single-Vendor Multicenter Study
Source: J Clin Med. 2026 Oct 27;15(21):8373. doi: 10.3390/jcm15218373 (PMC13764524; doi:10.3390/jcm15218373)
Supplement: Supplementary file 1 [file jcm-15-08373-s001.zip › Supplementary_Table_S4.pdf]

Supplementary Table S4. Effect of hepatic fibrosis on attenuation after adjustment for MRI-PDFF and association of the inter-protocol difference with fibrosis.

Panel A. Multivariable linear regression with the attenuation coefficient as the dependent variable.

	Conventional UGAP		WFUMB-based UGAP	
**Variable**	**Standardized β (95% CI)**	**p**	**Standardized β (95% CI)**	**p**
log MRI-PDFF	+0.733 (+0.675 to +0.792)	<0.001	+0.752 (+0.689 to +0.814)	<0.001
MRE at 60 Hz (kPa)	+0.028 (-0.021 to +0.077)	0.26	+0.033 (-0.020 to +0.086)	0.22
BMI	+0.012 (-0.083 to +0.108)	0.80	-0.080 (-0.182 to +0.022)	0.12
SCD	+0.136 (+0.046 to +0.227)	0.003	+0.151 (+0.054 to +0.248)	0.002

Note.– n = 552 patients with complete MRE, BMI and SCD data. All variables were standardized (mean 0, SD 1) before entry; β therefore expresses the change in attenuation coefficient in SD units per 1 SD change in the covariate. R^2 = 0.665 for Conventional UGAP and 0.617 for WFUMB-based UGAP. BMI and SCD were entered simultaneously in this model because both are determinants of the acoustic path length that the two protocols sample differently; collinearity was acceptable (variance inflation factor 3.84 for BMI and 3.47 for SCD). In a sensitivity analysis excluding SCD, MRE remained non-significant under both protocols (β = +0.022, p = 0.39 for Conventional UGAP; β = +0.026, p = 0.33 for WFUMB-based UGAP), and R^2 changed only marginally (0.660 and 0.611). After adjustment for MRI-PDFF, MRE was not independently associated with the attenuation coefficient under either protocol, and the point estimates were closely similar between protocols.

Panel B. Association between the inter-protocol difference (Conventional − WFUMB-based) and fibrosis measures.

Fibrosis measure	n	Spearman ρ	p
MRE (kPa)	561	+0.046	0.277
2D-SWE (kPa)	559	+0.075	0.076
FIB-4 index	565	-0.087	0.040

Note.– The inter-protocol difference did not differ across MRE-based fibrosis strata (Kruskal–Wallis H = 2.22, p = 0.33); the median difference was 0.020 dB/cm/MHz in F0–1, 0.020 dB/cm/MHz in F2–3 and 0.020 dB/cm/MHz in F4.

Abbreviations: BMI, body mass index; CI, confidence interval; FIB-4, Fibrosis-4 index; MRE, magnetic resonance elastography; MRI-PDFF, magnetic resonance imaging-derived proton density fat fraction; SCD, skin-to-capsule distance; SD, standard deviation; SWE, shear wave elastography; UGAP, ultrasound-guided attenuation parameter; WFUMB, World Federation for Ultrasound in Medicine and Biology.
